# The association between agranulocytosis and 30-day mortality in patients with hematological disorders and bloodstream infections: A retrospective cohort study

**DOI:** 10.1097/MD.0000000000045937

**Published:** 2026-02-13

**Authors:** Meng Zhou, Yuxia Jiang, Diehong Tao, Wenfei Zhou, Zhilu Chen, Huifang Jiang, Chuanyong Su

**Affiliations:** a Department of Hematology, Tongde Hospital of Zhejiang Province Affiliated to Zhejiang Chinese Medical University, Tongde Hospital of Zhejiang Province, Hangzhou, Zhejiang, China.

**Keywords:** 30-day mortality, agranulocytosis, bloodstream infections, hematological disorders

## Abstract

Agranulocytosis and bloodstream infections (BSIs) are common complications in patients with hematological disorders and they are life-threatening. This study aims to explore the association between agranulocytosis and 30-day mortality in individuals with hematological disorders and BSIs. In this retrospective cohort study, neutrophil levels were measured in patients presenting with fever and diagnosed with a hematological disorder at Tongde Hospital of Zhejiang Province between March 2018 and August 2023. The primary outcome was all-cause 30-day mortality. Multivariate Cox proportional hazard models were used to identify factors associated with survival among patients with hematological disorders and BSIs. Survival outcomes between agranulocytosis (neutrophils < 0.5 × 10^9^/L) and non-agranulocytosis (neutrophils ≥ 0.5 × 10^9^/L) were compared via the Kaplan–Meier method. Subgroup analyses were conducted, stratified by relevant effect covariates. The study included 113 (56.8%) males and 86 (43.2%) females, with a median age of 57.4 years. The overall 30-day mortality rate was 33.7% (67/199), with mortality rate of 26.1% (24/92) in the non-agranulocytosis group and 40.2% (43/107) in the agranulocytosis group (*P* = .036). After adjusting for potential confounders, a significant association between a 2% increase in the 30-day mortality rate and a 0.1 × 10^9^/L decrease in neutrophil levels was observed (95% confidence interval [CI]: 1.00–1.03, *P* = .006). Furthermore, agranulocytosis was associated with a 95% increase in 30-day mortality compared to non-agranulocytosis (95% CI: 1.10–3.46, *P* = .023). The findings from the subgroup and stratified analyses were consistent and robust. Agranulocytosis was positively associated with all-cause 30-day mortality in patients with hematological disorders and BSIs. It served as a significant early prognostic indicator for hematological patients who develop BSIs.

## 1. Introduction

Bloodstream infection (BSI)^[1]^ is a common and serious complication in patients with hematological disorders, attributed to compromised immune function, bone marrow suppression from chemotherapy or radiation therapy, and an increased risk of nosocomial infections associated with prolonged hospitalization. BSI represents a systemic infection which caused by various pathogens, including bacteria and fungi, and remains a leading contributor to morbidity and mortality, particularly in patients with hematological disorders. Prompt diagnose and treatment of BSI, along with the identification of pathogenic organisms, are crucial to prevent multiorgan failure and death.^[2]^

Numerous studies in patients with hematological disorders have demonstrated that neutropenia is an independent risk factor for BSI.^[3–5]^ The risk of infection rises as the neutrophil count decreases, with a further escalation in risk as the duration of neutropenia prolongs. Patients with agranulocytosis (absolute neutrophil count (ANC) < 0.5 × 10^9^/L) and those with granulocyte deficiency duration > 7 days have a significantly higher incidence of BSI, which potentially lead to sepsis even death.^[6,7]^ The crude mortality rate of BSI range from 34 to 50%, especially in cases with multi-drug resistance gram-negative organisms.^[8]^ Prolonged hospital stay, advanced disease, the presence of central venous catheter, and prior treatment with antibiotics or chemotherapy significantly associated with BSI in neutropenic patients with hematological diseases. Moreover, BSI in these patients can progress rapidly resulting in adverse outcomes, such as prolonged hospitalization, emotional, and social sequelae, which can undermine a family’s quality of life and lead to substantial medical costs.^[9]^

In the 1970s, gram-negative bacteria were the predominant pathogens isolated in BSI. However, by the 1980s, gram-positive bacteria became prevalent.^[10]^ This shift has been attributed to factors such as the increased use of intravascular catheters, more aggressive treatment leading to severe mucositis, and empiric antibiotic therapy against gram-negative bacteria in febrile neutropenic patients. Over the last decade, gram-negative bacteria as fatal causes of BSI have reemerged. Concurrently, the frequency of antimicrobial resistance has also increased.^[11]^

The relationship between agranulocytosis and 30-day mortality is a critical concern in patients with hematological disorders and BSI. To address this knowledge gap, we conducted a retrospective cohort study involving 199 participants with hematological disorders and BSI. Our primary objective of the study was to evaluate the association between agranulocytosis and 30-day mortality. The secondary objective was to investigate the combined effects of agranulocytosis and other interacting factors on 30-day mortality. Further investigation is needed to elucidate the underlying mechanisms. This study will provide us valuable insights and scientific guidance for future clinical work.

## 2. Material and methods

### 2.1. Study design and participants

This retrospective, single-center analysis included patients with hematological disorders who developed culture-confirmed BSI at the Hematology Department of Tongde Hospital of Zhejiang Province between March 1, 2018, and August 31, 2023. This study was approved by the Ethics Review Board of Tongde Hospital of Zhejiang Province. The requirement for informed consent was waived due to the retrospective nature of the study.

### 2.2. Data collection and definitions

Clinical data related to each BSI were collected from the hospital’s electronic medical record systems. For each participant, information on the date of BSI diagnosis, age, sex, height, weight, and placement of peripherally inserted central catheters were recorded. Laboratory data obtained at the time of first positive blood culture included white blood cell count, ANC, hemoglobin (Hb), platelets (PLT), C-reactive protein (CRP), albumin (ALB), globulin (GLB), alanine aminotransferase, urea, uric acid, creatinine, lactate dehydrogenase (LDH), procalcitonin (PCT). Pulmonary computed tomography imaging was performed within 48 hours following the onset of fever. Survival status was recorded via follow-up telephone calls. Microbiological results were obtained from the microbiology laboratory.

The ANC at the time of BSI diagnosis was used to classified eligible patients into 2 groups: an agranulocytosis (ANC < 0.5 × 10^9^/L) and non-agranulocytosis (ANC ≥ 0.5 × 10^9^/L) group. According to the US Centers for Disease Control and Prevention guidelines, BSI was defined as the isolation of a pathogen from a blood culture sample not secondary to infection at another site of the body.^[1]^

### 2.3. Statistical analysis

All statistical analyses were performed using R 4.0.2 (https://www.r-project.org, The R Foundation), and Free Statistics software v.1.9. Categorical data were presented as frequencies (percentages), while continuous data were reported as means ± standard deviation or as median with interquartile range ([IQR], quartile 1–quartile 3), as appropriate. Comparisons of categorical variables using χ^2^ tests and continuous variable using Mann–Whitney *U*-tests. Multivariate Cox regression analysis was used to assess the independent association between ANC levels and 30-day mortality. Models were adjusted for various covariates via an extended Cox model approach. The selection of covariates was based on previous findings and clinical relevance. Survival curves were plotted using the Kaplan–Meier method and assessed for statistical significance by the log-rank test. Subgroup analyses were stratified according to the relevant effect covariates. Effect modification within each subgroup was assessed using the likelihood ratio test, incorporating interaction terms between subgroup indicators and ANC. Interactions between subgroups were examined using likelihood ratio tests. A two-tailed *P* < .05 was considered statistically significant.

## 3. Results

### 3.1. Baseline characteristics of study participants by categories of ANC levels

**Figure 1.**
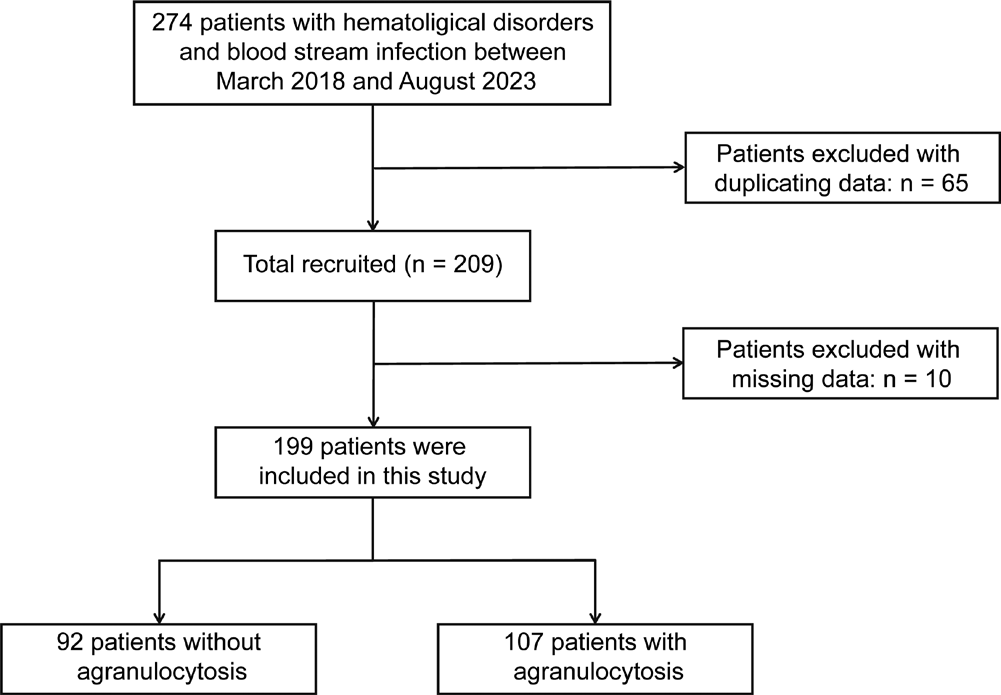
Flow chart of patient select.

Among the 274 patients with BSI, 199 were eligible for analysis (Fig. 1). Descriptive characteristics of the study population, categorized by agranulocytosis and non-agranulocytosis, are presented in Table 1. Within the eligible participants, 69 patients (34.7%) had B cell lymphoma with the highest proportion, followed by 48 patients (24.1%) with acute myeloid leukemia. Other subgroups were acute lymphoblastic leukemia (n = 23), T cell lymphoma (n = 22), multiple myeloma (n = 19), myelodysplastic syndrome/myeloproliferative neoplasms (n = 8) and non-neoplastic disease (n = 10). The median ANC level was 0.2 × 10^9^/L (IQR: 0–4.1 × 10^9^/L). The mean age of the patients was 57.4 years (range 14–92 years). Of the participants, 113 (56.8%) patients were male, 86 (43.2%) were female, and 67 (33.7%) patients died within 30 days after BSI diagnosis. Patients in the agranulocytosis group exhibited lower level of white blood cell count, Hb, PLT, ALB, urea, LDH, and higher levels of CRP compared with those with non-agranulocytosis (*P* < .001). The 30-day mortality rate was higher in the agranulocytosis group than in the non-agranulocytosis group (*P* = .036). Additionally, patients in the agranulocytosis group were younger than those in the non-agranulocytosis group (*P* < .001).

**Table 1:** Baseline characteristics of patients.

	Total	Non-agranulocytosis	Agranulocytosis	*P*-value
	n = 199	n = 92	n = 107	
Age (yr)	57.4 ± 19.2	64.8 ± 16.7	51.0 ± 18.9	<.001
Sex (n, %)				
Male	113 (56.8)	52 (56.5)	61 (57)	.945
Female	86 (43.2)	40 (43.5)	46 (43)	
BMI (kg/m^2^)	21.7 ± 3.3	22.4 ± 3.5	21.1 ± 3.0	.008
Primary disease (n, %)				
AML	48 (24.1)	8 (8.7)	40 (37.4)	<.001
ALL	23 (11.6)	3 (3.3)	20 (18.7)	
B-NHL	69 (34.7)	44 (47.8)	25 (23.4)	
T-NHL	22 (11.1)	7 (7.6)	15 (14)	
MDS/MPN	8 (4.0)	6 (6.5)	2 (1.9)	
MM	19 (9.5)	15 (16.3)	4 (3.7)	
Non-neoplastic disease	10 (5.0)	9 (9.8)	1 (0.9)	
Placement of PICC (n, %)				
No	47 (23.6)	31 (33.7)	16 (15)	.002
Yes	152 (76.4)	61 (66.3)	91 (85)	
Pulmonary infection (n, %)				
No	82 (41.2)	37 (40.2)	45 (42.1)	.793
Yes	117 (58.8)	55 (59.8)	62 (57.9)	
Time-to-positivity of blood cultures (d)	3.5 ± 0.9	3.7 ± 1.0	3.2 ± 0.6	<.001
Pathogen (n, %)				
Gram positive bacteria	49 (24.6)	29 (31.5)	20 (18.7)	.103
Gram negative bacteria	129 (64.8)	55 (59.8)	74 (69.2)	
Fungus	21 (10.6)	8 (8.7)	13 (12.1)	
WBC (×10^9^/L)	0.8 (0.2, 5.8)	5.8 (3.2, 9.7)	0.2 (0.1, 0.3)	<.001
ANC (×10^9^/L)	0.2 (0.0, 4.1)	4.3 (2.4, 7.1)	0.0 (0.0, 0.0)	<.001
Hb (g/L)	74.0 (65.0, 99.5)	98.0 (71.8, 120.0)	69.0 (62.0, 80.0)	<.001
PLT (×10^9^/L)	32.0 (13.0, 82.0)	78.0 (38.8, 138.2)	16.0 (6.0, 32.5)	<.001
CRP (mg/L)	59.1 (22.1, 137.4)	39.0 (10.9, 108.5)	77.8 (33.4, 185.0)	<.001
ALB (g/L)	33.5 (28.7, 37.5)	35.0 (29.3, 38.3)	32.6 (28.5, 36.2)	.02
GLB (g/L)	23.6 (19.7, 28.2)	24.4 (20.5, 29.7)	22.8 (19.4, 26.5)	.052
ALT (U/L)	23.0 (14.0, 46.5)	19.0 (14.0, 38.5)	25.0 (15.5, 63.5)	.043
Urea (mmol/L)	6.8 (5.0, 8.8)	7.0 (5.1, 9.3)	6.4 (4.9, 8.5)	.415
UA (µmol/L)	212.0 (148.5, 310.5)	266.0 (197.2, 371.5)	179.0 (129.0, 230.0)	<.001
Cr (µmol/L)	57.0 (44.0, 80.5)	66.0 (48.0, 88.2)	51.0 (42.0, 75.0)	.003
LDH (U/L)	218.0 (151.0, 323.5)	243.0 (189.0, 378.2)	171.0 (129.0, 275.5)	<.001
PCT (ng/mL)	0.5 (0.2, 2.0)	0.4 (0.1, 1.1)	0.6 (0.2, 2.8)	.076
Survival time (d)	107.0 (12.0, 409.0)	150.0 (20.5, 587.5)	68.0 (6.0, 347.0)	.013
30-d mortality (n, %)				
No	132 (66.3)	68 (73.9)	64 (59.8)	.036
Yes	67 (33.7)	24 (26.1)	43 (40.2)	

Data presented are mean ± SD, median (IQR), or N (%).

ALB = albumin, ALL = acute lymphocytic leukemia, ALT = alanine aminotransferase, AML = acute myeloid leukemia, ANC = absolute neutrophil count, BMI = body mass index, Cr = creatinine, CRP = C-reactive protein, GLB = globulin, Hb = hemoglobin, LDH = lactate dehydrogenase, MDS = myelodysplastic syndrome, MM = multiple myeloma, MPN = myeloproliferative neoplasms, NHL = non-Hodgkin’s lymphoma, PCT = procalcitonin, PICC = peripherally inserted central catheter, PLT = platelet, UA = uric acid, WBC = white blood cell.

### 3.2. Distribution of pathogens

**Figure 2.**
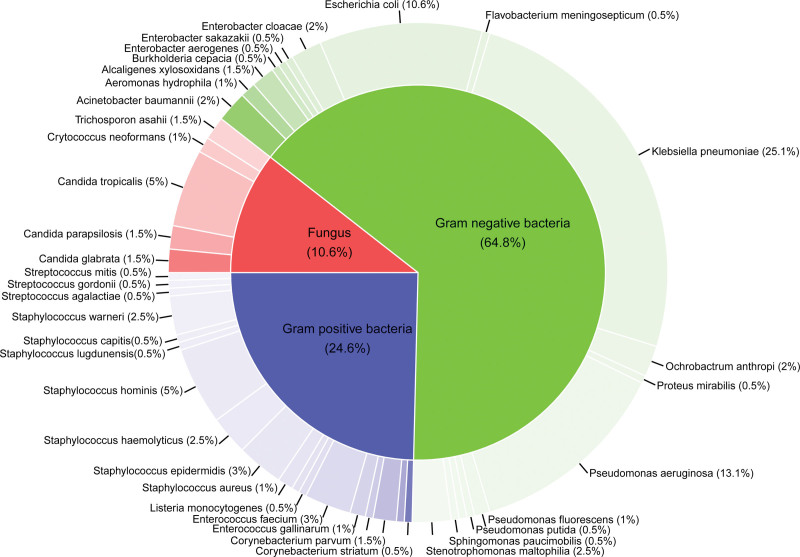
Pie chart for distribution of pathogens.

Among the 199 BSI cases, gram-negative infections were the most prevalent (n = 129, 64.8%), followed by gram-positive infections (n = 49, 24.6%) and fungal infections (n = 21, 10.6%) (Fig.2, Table SI, Supplemental Digital Content, https://links.lww.com/MD/Q620). The most common isolated gram-negative bacterium was *Klebsiella pneumoniae* (n = 50, 25.13%), followed by *Pseudomonas aeruginosa* (n = 26, 13.07%) and *Escherichia coli* (n = 21, 10.55%). Among gram-positive bacteria, *Staphylococcus hominis* was the most frequently isolated (n = 10, 5.03%), followed by *Staphylococcus epidermidis* (n = 6, 3.02%) and *Enterococcus faecium* (n = 6, 3.02%).

### 3.3. Association between ANC and 30-day mortality

Univariate analysis of risk factors associated with 30-day mortality in patients with hematological disorders and BSI is summarized in Table 2. It showed hazard ratios (HR) and 95% confidence intervals (CIs) for the risk among patients, body mass index (BMI), pulmonary infection, agranulocytosis, ANC, Hb, PLT, CRP, ALB, GLB, urea, LDH, and PCT were significantly associated with 30-day mortality (*P* < .05).

**Table 2:** Univariate analysis of risk factor associated with 30-d mortality in patients with hematologic disorders and blood stream infection.

	HR (95% CI)	*P*-value
Age	1.00 (0.99–1.01)	.919
Sex		
Male	Ref.	.896
Female	1.03 (0.64–1.67)	
BMI	0.86 (0.80–0.93)	<.001
Placement of PICC		
No	Ref.	.852
Yes	0.95 (0.54–1.66)	
Pulmonary infection		
No	Ref.	<.001
Yes	3.43 (1.87–6.29)	
Time-to-positivity of blood cultures	0.81 (0.59–1.11)	.190
Agranulocytosis		
No	Ref.	.023
Yes	1.78 (1.08–2.94)	
Pathogen		
Fungus	Ref.	.453
Gram negative bacteria	0.65 (0.32–1.29)	
Gram positive bacteria	0.61 (0.28–1.35)	
WBC	0.96 (0.91–1.01)	.136
ANC	0.88 (0.80–0.96)	.006
Hb	0.97 (0.96–0.98)	<.001
PLT	0.99 (0.98–0.99)	<.001
CRP	1.01 (1.00–1.01)	<.001
ALB	0.86 (0.82–0.89)	<.001
GLB	0.96 (0.92–1.00)	.044
ALT	1.00 (0.99–1.01)	.491
Urea	1.12 (1.07–1.16)	<.001
UA	1.00 (0.99–1.00)	.895
Cr	0.99 (0.99–1.00)	.476
LDH	1.00 (1.00–1.00)	<.001
PCT	1.02 (1.00–1.05)	.042

ALB = albumin, ALT = alanine aminotransferase, ANC = absolute neutrophil count, BMI = body mass index, CI = confidence interval, Cr = creatinine, CRP = C-reactive protein, GLB = globulin, Hb = hemoglobin, HR = hazard ratios, LDH = lactate dehydrogenase, PCT = procalcitonin, PICC = peripherally inserted central catheter, PLT = platelet, UA = uric acid, WBC = white blood cell.

In the extended multivariate Cox models (Table 3), HR of ANC (per 0.1 × 10^9^/L decrease) were consistently significant across all 3 models (HR range: 1.01–1.02). The risk for 30-day mortality was higher increased in the agranulocytosis group, with a non-adjusted HR of 1.78 (95% CI: 1.08–2.94), compared to the non-agranulocytosis group. After adjusting for covariates selected from univariate analysis (Table 2), patients with agranulocytosis demonstrated a 95% increase in 30-day mortality rate (HR = 1.95, 95% CI: 1.10–3.46, *P* = .023, Model Ⅲ), compared to those with non-agranulocytosis. These statistical results remained robust across all models (Table 3).

**Table 3:** Multivariate cox regression for agranulocytosis on 30-d mortality of patients with hematologic disorders and blood stream infection.

Variable	Not-adjusted model		Model I		Model II		Model III	
	HR (95% CI)	*P*-value	HR (95% CI)	*P*-value	HR (95% CI)	*P*-value	HR (95% CI)	*P*-value
ANC	1.01 (1.00–1.02)	.006	1.02 (1.01–1.03)	.003	1.01 (1.00–1.03)	.009	1.02 (1.00–1.03)	.006
Binary variable								
Non-agranulocytosis	Ref.	.023	Ref.	.012	Ref.	.039	Ref.	.023
Agranulocytosis	1.78 (1.08–2.94)		2 (1.17–3.44)		1.83 (1.03–3.23)		1.95 (1.10–3.46)	

ANC was entered as a continuous variable per 0.1 × 10^9^/L decrease. Data presented are HRs and 95% CIs. Model I model adjusts for age and sex; Model II model adjusts for adjust I + BMI + placement of PICC + pulmonary infection + time-to-positivity of blood cultures; Model III model adjusts for adjust II + CRP + PCT + ALB + GLB + urea + LDH.

ALB = albumin, ANC = absolute neutrophil count, BMI = body mass index, CI = confidence interval, CRP = C-reactive protein, GLB = globulin, HR = hazard ratios, LDH = lactate dehydrogenase, PCT = procalcitonin, PICC = peripherally inserted central catheter.

**Figure 3.**
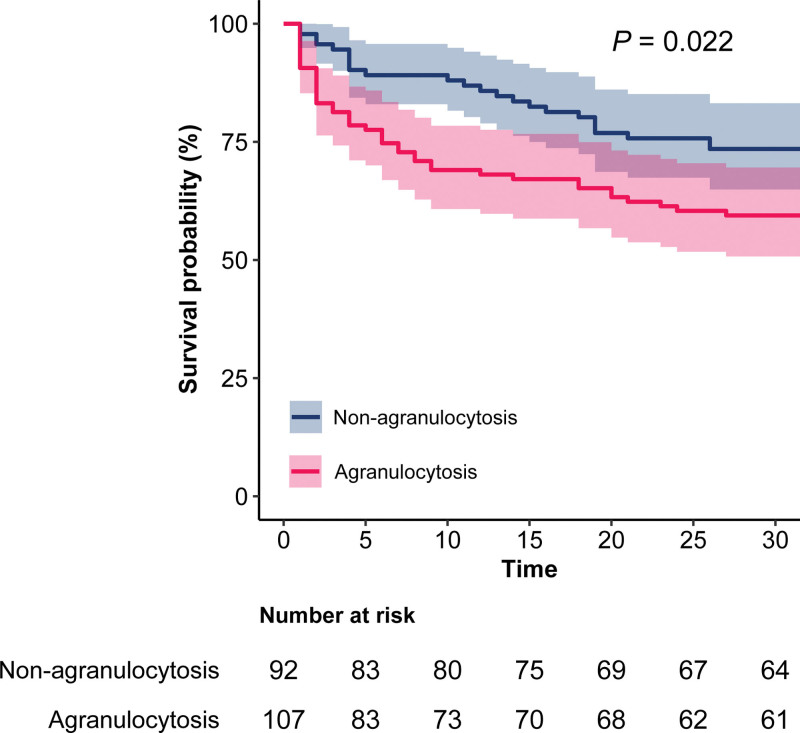
Kaplan–Meier survival curves for 30-d of patients with hematological disorders and bloodstream infection.

The Kaplan–Meier curve revealed that patients in the agranulocytosis group had a higher 30-day mortality rate than those in the non-agranulocytosis group (log-rank test: *P* = .022, Fig. 3).

### 3.4. Subgroup analyses

**Figure 4.**
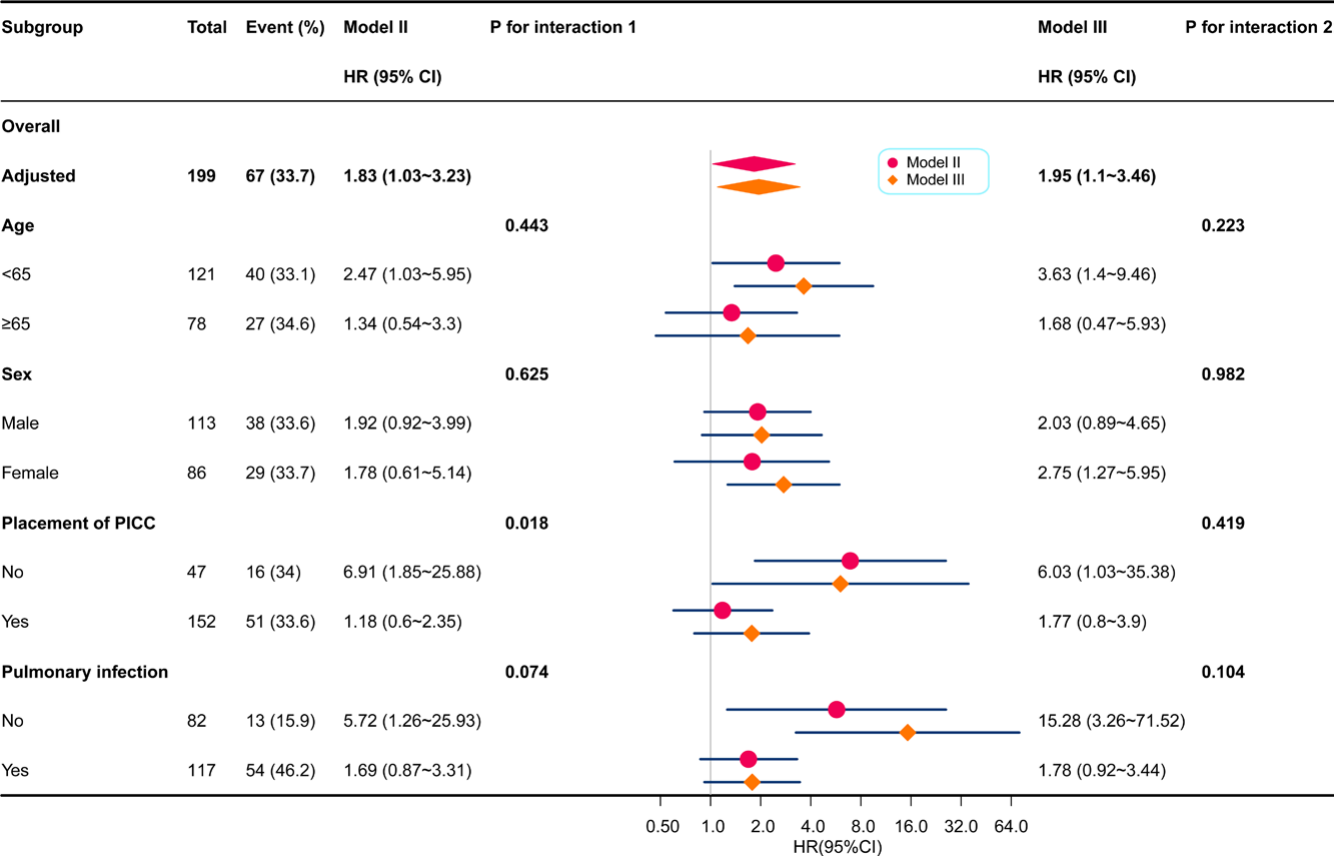
Subgroup analysis of the association between agranulocytosis and 30-d mortality of patient with hematological disorders and bloodstream infection. Model II model adjusts for age + sex + BMI + placement of PICC + pulmonary infection + time-to-positivity of blood cultures; Model III model adjusts for Model II + CRP + PCT + ALB + GLB + urea + LDH. ALB = albumin, BMI = body mass index, CRP = C-reactive protein, GLB = globulin, LDH = lactate dehydrogenase, PICC = peripherally inserted central catheter.

To investigate whether the association between agranulocytosis and 30-day mortality in patients with hematological disorders and BSI varied across different subgroups, stratified and interactive analyses were used (Fig. 4). Model Ⅱ was adjusted for age, sex, BMI, placement of peripherally inserted central catheter, pulmonary infection, and time-to-positivity of blood cultures. Model Ⅲ adjusted for the variables in Model Ⅱ and included CRP, PCT, ALB, GLB, urea, and LDH. No stratification variables showed an interaction between agranulocytosis and 30-day mortality in either model.

## 4. Discussion

BSI represents a significant challenge in the field of infectious diseases globally. The characteristics of the underlying disease, coupled with the influence of chemotherapy or immunosuppressive treatments, contribute to patients with hematological disorders experiencing the highest incidence of infection. Among these patients, the incidence of BSI is estimated to range from 11 to 38%,^[12]^ with a mortality rate of 6 to 36%.^[13,14]^ When the pathogen is multi-drug resistant, the mortality rate is significantly increased.^[15]^ In patients with agranulocytosis, the mortality rate from BSIs is even higher, ranging from 7.1 to 42%.^[16,17]^ In the study, the overall mortality rate of patients was 33.7%, with a significantly higher mortality rate of 40.2% observed in patients with agranulocytosis compared to 26.1% in those without agranulocytosis (*P* = .036), consistent with the findings of previous studies.^[16,17]^

According to data from the China Antimicrobial Surveillance Network and previous studies, the predominant pathogenic species in BSI patients are gram-negative bacteria, mainly consisting of Enterobacteriaceae (i.e., *E. coli*, *K. pneumoniae*, *Enterobacter cloacae*), and non-fermentative bacteria (i.e., *P. aeruginosa*, *Acinetobacter baumannii*, *Stenotrophomonas maltophilia*),^[18,19]^ a finding corroborated by our study. In this study, the infection rate of gram-negative bacteria was 64.8%, with *K. pneumoniae*, *P. aeruginosa,* and *E. coli* being the most common pathogens. These findings are consistent with previous studies.^[20]^ The most prevalent gram-positive bacteria were *S. hominis*, *S. epidermidis*, and *E. faecium*, which is partially consistent with previous findings.^[21]^ The discrepancies observed between studies may be attributed to geographical variations, changes in antimicrobial susceptibility profiles of the pathogens, or the use of empirical anti-infective therapy in patients.

Given the high morbidity and mortality associated with BSIs, extensive research has focused on risk factors for infection and prognosis in patients with BSIs. Statistically significant differences were observed between patients with and without BSI in terms of age, neutrophil count, granulocyte deficiency time, invasive procedures, types of anti-infective drugs employed, chemotherapy treatment, and glucocorticoid use (*P* < .05). Further binary logistic regression analysis demonstrated that age > 60, neutrophil value ≤ 0.2 × 10^9^/L, granulocyte deficiency duration > 7 days, use of more than 2 types of anti-infective drugs, chemotherapy, and glucocorticoid use were independent risk factors for BSIs in patients with hematological diseases.^[22]^ The findings of this study suggested that agranulocytosis may act as a predisposing factor for BSIs. Additionally, other studies have identified granulocyte deficiency lasting more than 7 days, central catheterization, carbapenem resistance, and inappropriate initial empirical treatment as independent risk factors for BSI mortality in patients with malignant hematological diseases.^[23]^ In our study, BSI patients with agranulocytosis had a higher 30-day mortality rate than those without agranulocytosis (40.2% vs 26.1%, *P* = .036). Furthermore, regression analysis revealed that BMI, pulmonary infection, granulocyte deficiency, Hb, platelet count, CRP, ALB, urea, LDH, and PCT were independent risk factors for all-causes 30-day mortality. These findings demonstrated the significant association between agranulocytosis and 30-day mortality in patients. Limitations of the study include the study design itself, and the potential influence of social, genetic, and environmental factors.

## 5. Conclusion

In conclusion, the findings of our study demonstrated that patients in the agranulocytosis group had a significantly higher 30-day mortality compared to those without agranulocytosis. The study showed that BMI, pulmonary infection, agranulocytosis, ANC, Hb, PLT, CRP, ALB, GLB, urea, LDH, and PCT levels were all significantly associated with 30-day mortality. Furthermore, agranulocytosis played as an independent risk factor for all-causes 30-day mortality. The results of subgroup analyses further reinforce the reliability of our findings. Agranulocytosis serves as a significant early prognostic indicator for hematological patients who develop BSIs. However, there were several limitations in this study. Firstly, due to the retrospective nature and single-center design, it may be subject to indication bias. Secondly, data of antibiotic resistance and therapeutic regimens, as well as their potential impact on mortality, were not investigated. Thus, future multi-center studies incorporating more influencing factors are necessary.

## Acknowledgments

We gratefully thank Jie Liu, PhD (Department of Vascular and Endovascular Surgery, Chinese PLA General Hospital), for his helpful review and comments regarding the manuscript.

## Author contributions

**Data curation:** Meng Zhou, Yuxia Jiang, Wenfei Zhou, Zhilu Chen.

**Formal analysis:** Meng Zhou, Diehong Tao, Chuanyong Su.

**Investigation:** Huifang Jiang.

**Project administration:** Chuanyong Su.

**Validation:** Zhilu Chen.

**Writing – original draft:** Meng Zhou, Yuxia Jiang.

**Writing – review & editing:** Chuanyong Su.

## Supplementary Material


