# Supplementary material for: The association between agranulocytosis and 30-day mortality in patients with hematological disorders and bloodstream infections: A retrospective cohort study
Source: Medicine (Baltimore). 2026 Feb 13;105(7):e45937. doi: 10.1097/MD.0000000000045937 (PMC12908854; doi:10.1097/MD.0000000000045937)
Supplement: Supplementary file 1 [file medi-105-e45937-s001.docx]

**Supplementary Table I. Distribution of pathogens**

| **Pathogen** | **Number (%)** |
| --- | --- |
| **Gram-positive bacteria** | **49 (24.62)** |
| Staphylococcus hominis | 10 (5.03) |
| Staphylococcus epidermidis | 6 (3.02) |
| Enterococcus faecium | 6 (3.02) |
| Staphylococcus haemolyticus | 5 (2.51) |
| Staphylococcus warneri | 5 (2.51) |
| Corynebacterium parvum | 3 (1.51) |
| Staphylococcus aureus | 2 (1.01) |
| Enterococcus gallinarum | 2 (1.01) |
| Streptococcus agalactiae | 1 (0.50) |
| Streptococcus gordonii | 1 (0.50) |
| Streptococcus mitis | 1 (0.50) |
| Staphylococcus lugdunensis | 1 (0.50) |
| Staphylococcus capitis | 1 (0.50) |
| Enterococcus avium | 1 (0.50) |
| Micrococcus luteus | 1 (0.50) |
| Listeria monocytogenes | 1 (0.50) |
| Bacillus | 1 (0.50) |
| Corynebacterium striatum | 1 (0.50) |
| **Gram-negative bacteria** | **129 (64.82)** |
| Klebsiella pneumoniae | 50 (25.13) |
| Pseudomonas aeruginosa | 26 (13.07) |
| Escherichia coli | 21 (10.55) |
| Stenotrophomonas maltophilia | 5 (2.51) |
| Enterobacter cloacae | 4 (2.01) |
| Acinetobacter baumannii | 4 (2.01) |
| Ochrobactrum anthropi | 4 (2.01) |
| Alcaligenes xylosoxidans | 3 (1.51) |
| Aeromonas hydrophila | 2 (1.01) |
| Pseudomonas fluorescens | 2 (1.01) |
| Sphingomonas paucimobilis | 1 (0.50) |
| Pseudomonas putida | 1 (0.50) |
| Enterobacter sakazakii | 1 (0.50) |
| Enterobacter aerogenes | 1 (0.50) |
| Burkholderia cepacia | 1 (0.50) |
| Flavobacterium meningosepticum | 1 (0.50) |
| Proteus mirabilis | 1 (0.50) |
| Salmonella enteritidis | 1 (0.50) |
| **Fungus** | **21 (10.56)** |
| Candida tropicalis | 10 (5.03) |
| Trichosporon asahii | 3 (1.51) |
| Candida glabrata | 3 (1.51) |
| Candida parapsilosis | 3 (1.51) |
| Crytococcus neoformans | 2 (1.01) |
